# Recent Advances on the Role of GSK3β in the Pathogenesis of Amyotrophic Lateral Sclerosis

**DOI:** 10.3390/brainsci10100675

**Published:** 2020-09-26

**Authors:** Hyun-Jun Choi, Sun Joo Cha, Jang-Won Lee, Hyung-Jun Kim, Kiyoung Kim

**Affiliations:** 1 Department of Integrated Biomedical Science, Soonchunhyang University, Cheonan 31151, Korea; chj5913@sch.ac.kr; 2 Department of Medical Science, Soonchunhyang University, Asan 31538, Korea; cktjswn92@sch.ac.kr; 3 Department of Integrated Bio-industry, Sejong University, Seoul 05006, Korea; wintrelove@sejong.ac.kr; 4 Dementia Research Group, Korea Brain Research Institute (KBRI), Daegu 41068, Korea; kijang1@kbri.re.kr; 5 Department of Medical Biotechnology, Soonchunhyang University, Asan 31538, Korea

**Keywords:** amyotrophic lateral sclerosis, GSK3β, neurodegenerative disease

## Abstract

Amyotrophic lateral sclerosis (ALS) is a common neurodegenerative disease characterized by progressive motor neuron degeneration. Although several studies on genes involved in ALS have substantially expanded and improved our understanding of ALS pathogenesis, the exact molecular mechanisms underlying this disease remain poorly understood. Glycogen synthase kinase 3 (GSK3) is a multifunctional serine/threonine-protein kinase that plays a critical role in the regulation of various cellular signaling pathways. Dysregulation of GSK3β activity in neuronal cells has been implicated in the pathogenesis of neurodegenerative diseases. Previous research indicates that GSK3β inactivation plays a neuroprotective role in ALS pathogenesis. GSK3β activity shows an increase in various ALS models and patients. Furthermore, GSK3β inhibition can suppress the defective phenotypes caused by SOD, TDP-43, and FUS expression in various models. This review focuses on the most recent studies related to the therapeutic effect of GSK3β in ALS and provides an overview of how the dysfunction of GSK3β activity contributes to ALS pathogenesis.

## 1. Introduction

Amyotrophic lateral sclerosis (ALS) is a fatal neurodegenerative disease characterized by progressive and selective degeneration of the upper and lower motor neurons in the brain and spinal cord [1,2,3]. The majority of reported ALS cases are classified into two categories: sporadic ALS (sALS) and familial ALS (fALS). Approximately 90% of patients suffer from sALS and do not have a family history of the disease. The remaining 10% of patients suffer from fALS, which is strongly associated with the presence of a family history and genetic cause of the disease [1]. Since the initial discovery of *copper–zinc superoxide dismutase (SOD1)* as a causal gene for ALS, numerous genes such as *TAR DNA-binding protein 43* (*TDP-43*), *fused in sarcoma* (*FUS*), *TATA-binding protein-associated factor 15* (*TAF15*), and *chromosome 9 open reading frame 72 (C9orf72*), have been found to be associated with fALS and sALS [4,5,6,7,8,9,10]. Various RNA/DNA-binding proteins share structural and functional properties that contribute to the pathogenesis of ALS. Although several studies on ALS-causing genes have substantially expanded and improved our understanding of ALS pathogenesis, the underlying molecular mechanisms of the disease remain poorly understood. Recent studies have discovered various genetic susceptibility factors, using several in vivo and in vitro models, that could explain the underlying mechanisms involved in ALS pathogenesis. Understanding these mechanisms would be immensely helpful for developing new drug targets for ALS treatment.

Glycogen synthase kinase 3 (GSK3) is a cellular serine/threonine protein kinase that is involved in glycogen metabolism. GSK3 is also a phosphorylating and an inactivating agent of glycogen synthase [11,12]. GSK3 is encoded by two paralogous genes, *GSK3α* and *GSK3β*. GSK3β is the more studied and better characterized GSK3 isoform because of its predominant expression in the majority of cells and tissues. The specific function of GSK3α is less known. GSK3β is expressed ubiquitously, but the only cells currently known to express GSK3α predominantly, compared to GSK3β, are spermatozoa [13]. Conditional knockout of *GSK3α* in developing testicular germ cells in mice results in male infertility [14]. Other studies reported a role for GSK3α in central nervous system functioning and possible involvement in the development of psychiatric disorders [15]. GSK3β participates in a variety of critical cellular processes, such as glycogen metabolism, gene transcription, apoptosis, and microtubule stability [16]. Dysfunction of GSK3β is implicated in a variety of diseases, including type 2 diabetes and cancer. Recent studies have also suggested a possible role of GSK3β in neurodegenerative diseases, such as Parkinson’s disease (PD) and Alzheimer’s disease (AD) [16,17]. As it inhibits several common pathogenic pathways in neurodegenerative diseases, GSK3β could be a potential target for the development of novel therapeutics for neurodegenerative diseases. Recent studies suggest that GSK3β may have a definitive role in the pathogenesis of ALS. In this review, we present the evidence from these GSK3β studies in ALS and summarize the data into two categories: in vitro and in vivo models.

## 2. Role of GSK3β Signaling in Neurons

There are two types of GSK3 isoforms, GSK3α and GSK3β, which have an overall 85% sequence identity and 95% homology in the kinase domains [18]. Moreover, it has been reported that both GSK3 isoforms are highly expressed in the brain and spinal cord [19]. There are two splice variants of GSK3β in rodents and humans, short-form (GSK3β1) and long-form (GSK3β2). GSK3β contains a 13 amino acid insert in the catalytic domain due to alternative splicing [19]. In contrast to ubiquitously expressed human GSK3β1, GSK3β2 is specifically expressed in the developing nervous system [19]; human GSK3β2 is expressed only in neurons during the differentiation stage and not in glial cells, whereas human GSK3β1 is expressed in glial cells [20]. Furthermore, recent studies suggest that the upregulation of human GSK3β2 in PC12 cells induces nerve growth factor to differentiate into a neuronal phenotype, playing a specific role in neuronal morphogenesis [20,21,22]. These findings suggest that the GSK3β isoforms may have a significant role in the development of neurons in the nervous system. Neuronal progenitor proliferation and differentiation are regulated by multiple extracellular and intracellular signaling pathways that are closely associated with GSK3β [23,24,25]. Previous studies revealed that GSK3 signaling is an essential mediator of homeostatic controls that regulate neural progenitors during mammalian brain development. Inactivation of GSK3β in mouse neural progenitors resulted in the hyperproliferation of neural progenitors. Moreover, the generation of both intermediate neural progenitors and postmitotic neurons was markedly suppressed [24]. GSK3β regulates the stability of various proteins through the ubiquitin–proteasome system [26,27]. GSK3β controls progenitor proliferation or differentiation by regulating the levels of transcription regulators involved in neurogenesis, such as β-catenin in Wnt signaling, Gli in Sonic Hedgehog (Shh) signaling, and c-Myc in fibroblast growth factor signaling in the nervous system [24,28,29]. In addition, GSK3β is an essential regulator of microtubule–cytoskeleton reorganization, neuronal polarity, and neuronal migration by phosphorylating key microtubule-associated proteins [30,31,32], such as microtubule plus-end-tracking proteins (+TIPs) [33], collapsing response mediator protein-2 (CRMP-2) [34], adenomatous polyposis coli (APC) [35], cytoplasmic linker associated proteins (CLASPs) [36], microtubule-associated protein 1B (MAP1B) [37], and tau [38]. APC and CLASP promote microtubule stability. However, phosphorylation of APC and CLASPs by GSK3β induces decreased activity and leads to the destabilization of microtubules in neurons [36,39]. Therefore, polarized deposition of polarity proteins underlies asymmetric cell division, which is necessary for the neurogenic division of neural progenitors. Indeed, the polarized apical concentrations of markers, including APC, cadherin, and end-binding 1 (EB1) were found to be significantly reduced in the cortex of GSK3β-deleted mice [24].

In addition, GSK3β is associated with the formation of neuronal morphology, including axonal growth, dendritic branching, and the development of synapses [40]. Inhibition of GSK3β activity impairs axon formation and disturbs polarity development by leading to the formation of multiple axon-like processes in neurons [41,42]. Furthermore, the genetic activation of GSK3β activity results in a shrunken form of dendrites, whereas inhibition of GSK3β activity promotes dendritic growth in vivo [43].

GSK3β activity affects neuroplasticity and neurotransmission. Glutamatergic synapses are the main excitatory synapses in the brain and consist of glutamate localized inside the presynaptic vesicles and glutamate receptors on the postsynaptic membrane. GSK3β regulates the interaction between two major forms of synaptic plasticity at glutamatergic synapses, in an N-methyl-D-aspartate (NMDA)-dependent long-term potentiation (LTP) and a long-term depression (LTD) manner. During LTP, the activation of NMDA receptors causes the inhibition of GSK3β activity through the phosphatidylinositol 3-kinase (PI3-K)/Akt pathway. The inhibition of GSK3β is necessary for the induction of LTP, the process underlying new memory formation, whereas the action of protein phosphatase 1 (PP1) in LTD causes an increase in GSK3β activity, which is related to the downsizing of synapses and decreased excitability of neurons. Thus, GSK3β is crucial for the initiation of NMDA-induced LTD in neurons [44,45]. Furthermore, the inhibition of GSK3β-mediated phosphorylation of gephyrin-Ser270, which is a scaffolding protein, induces GABAergic synaptogenesis [43,46].

In another function, GSK3β plays a key role in regulating metabolic proteins, intermediary metabolism, and mitochondrial function. AMP-activated protein kinase (AMPK) is a major factor that regulates cellular energy homeostasis by inhibiting anabolic processes and activating catabolic ones [47]. GSK3β constitutively interacts with the AMPK complex through the β subunit and phosphorylates the α subunit of AMPK. This phosphorylation enhances the accessibility of the activation loop of the subunit to phosphatases, thereby inhibiting the AMPK kinase activity [48]. Furthermore, the PI3-K/Akt pathway, which is a major anabolic signaling pathway, enhances GSK3β-dependent phosphorylation of the α subunit. This suggests that GSK3β-dependent AMPK inhibition is critical for cells to enter an anabolic state [48]. GSK3β is enriched in many neurons that critically depend on mitochondrial function. GSK3β also acts as a negative regulator of mitochondrial energy metabolism by inhibiting the activity of pyruvate dehydrogenase, which attenuates mitochondrial activity [49]. Mitochondrial dynamics are important for the maintenance of mitochondrial homeostasis [50,51]. GSK3β regulates mitochondrial dynamics via phosphorylation of dynamin-related protein 1 (Drp1), which regulates mitochondrial fission [52]. Mitochondrial fusion or fission is regulated by the phosphorylation site of Drp1, which is mediated by GSK3β [53]. GSK3β also regulates mitochondrial metabolism through PGC-1α, a transcriptional coactivator and master regulator of mitochondrial function. GSK3β regulates PGC-1α stability through phosphorylation, which can be recognized by the E3 ubiquitin ligase [54]. Several studies have shown that GSK3β regulates mitochondrial energy metabolism in the neurons and glia [55]. Moreover, GSK3β inhibition increases mitochondrial respiration and membrane potential, and alters NAD(P)H metabolism in the neurons. Moreover, GSK3β inhibition alters PGC-1α protein stability, localization, and activity. These studies support the idea that GSK3β may be important in neuronal metabolic integrity [55].

**Figure 1:**
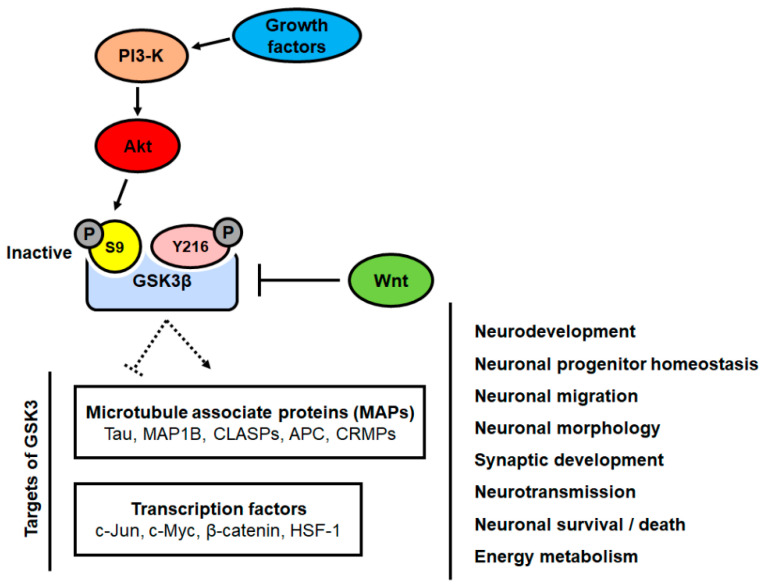
Role and regulation of glycogen synthase kinase 3 (GSK3) in the neuron. The Wnt and phosphatidylinositol 3-kinase (PI3-K)/Akt signaling pathways induce the inhibition of GSK3 activity by phosphorylating the serine 9 residue. This leads to subsequent regulation of its target proteins. GSK3 and its target proteins regulate a variety of biological processes, including neurodevelopment, neuronal migration, neurotransmission, neuronal cell death, and energy metabolism in the neurons.

Thus, functional studies of GSK3β have shown that GSK3β plays key roles in many fundamental processes in the neurons during neurodevelopment, including neuronal progenitor homeostasis, neuronal migration, neuronal morphology, synaptic development, and neurotransmission. Furthermore, it plays a crucial role in the energy metabolism of neurons (Figure 1).

## 3. Role of GSK3β in Neurodegenerative Diseases

GSK3 is a serine/threonine-protein kinase involved in glycogen metabolism and is also a phosphorylating and an inactivating agent of glycogen synthase [11,12]. It has two isoforms, GSK3α and GSK3β. GSK3α is predominantly present in the nucleus, and GSK3β is present in the cytoplasm. GSK3β plays an important role in the brain and participates in a variety of cellular processes, such as glycogen metabolism, gene transcription, apoptosis, and microtubule stability [16]. GSK3β is classified as a predominantly cytoplasmic enzyme but also localizes to three cellular compartments: the cytosol, the nucleus, and mitochondria [56]. The enzymatic activity of GSK3β is regulated by phosphorylation and depends on the phosphorylation of certain sites. This occurs through phosphorylation of the tyrosine 216 residue (Tyr216) located in the kinase region and is inactivated via phosphorylation of the amino-terminal serine 9 residue (Ser9) [16]. GSK3β is also involved in diverse cell signaling pathways. GSK3β activity is inhibited by the Wnt signaling pathway and is also negatively regulated by the PI3-K/Akt pathway (Figure 1). Additionally, activated GSK3β inhibits heat shock transcription factor-1 (HSF-1), affects the mitochondrial death pathway, and releases cytochrome *c* from the mitochondria. Released cytochrome *c* activates caspase-3 and induces apoptosis [57]. Dysfunction of GSK3β has been implicated in a variety of neurodegenerative diseases, such as PD and AD [16,17]. Many studies have shown that GSK3β is activated by amyloid-beta (Aβ) in AD, which eventually induces neuronal cell death and axonal transport defects by hyperphosphorylation of tau [58]. 

GSK3β also plays a critical role in the pathogenic mechanisms of PD and AD. α-synuclein is the most important factor in the pathogenesis of PD. Studies have shown that α-synuclein can also activate GSK3β. GSK3β inactivation decreases the protein level of α-synuclein, which in turn decreases α-synuclein-induced cell death in a cellular model of PD. Furthermore, activated GSK3β mediates the neurotoxic effects of tau hyperphosphorylation in AD [59]. Together, these findings suggest that inactivation of the GSK3 pathway may be an important therapeutic strategy for the treatment of PD and AD. In patients who have suffered a stroke, treatment with GSK3β inhibitors promotes neurovascular remodeling and improves ischemia. Recent studies have shown that GSK3β is closely associated with neuronal diseases [60]. Although the role of GSK3β in the pathogenesis of ALS remains poorly understood, recent evidence implicates GSK3β dysfunction in ALS models. Further studies are needed to determine the exact molecular mechanisms through which GSK3β affects ALS pathogenesis.

## 4. Studies Exploring the Role of GSK3β in In Vivo Models of ALS

Recent studies have reported that GSK3β is involved in the pathogenesis of ALS. Hu et al. reported that GSK3β expression and cytosolic levels of phospho-Y216 GSK3β increased in the spinal cord and frontal and temporal cortices of ALS patients [61]. In addition, Yang et al. demonstrated that the expression of GSK3β and phospho-Y216 GSK3β increased in patients with ALS compared to that in the controls, and that GSK3β and phospho-GSK3β immunoreactive neurons were mainly located in the frontal cortex and hippocampus of ALS patients [62]. These results show that GSK3β plays a critical role in ALS pathogenesis. Further studies are needed to determine the molecular mechanism of GSK3β in ALS pathogenesis. To investigate the molecular mechanism of GSK3β in ALS, many studies have been conducted using animal models. A genetic mutation in *SOD1* is one of the most common and important causes of ALS, accounting for 23% of fALS causes and approximately 7% of sALS causes worldwide [63]. A number of animal models of ALS are based on this gene. 

**Figure 2:**
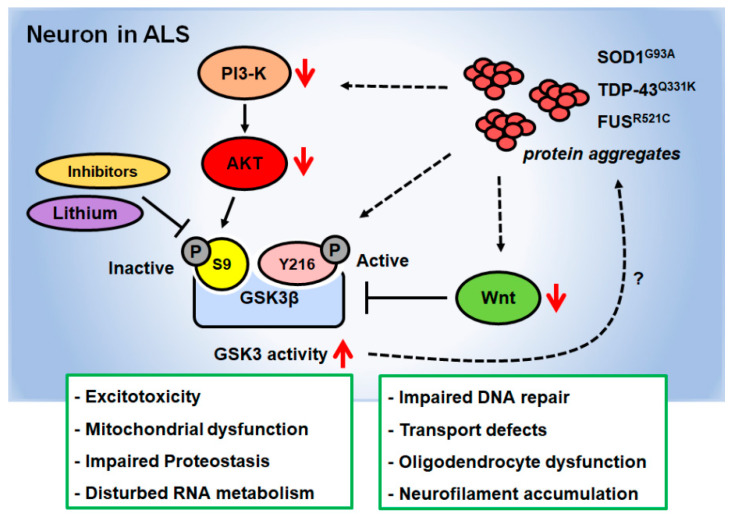
Abnormal regulation of the GSK3 activity in amyotrophic lateral sclerosis (ALS). A number of studies have found the inhibition of the PI3K/Akt and Wnt pathway in injured motor neurons of ALS pathological condition. This leads to abnormal activation of the GSK3 signaling in ALS neurons. The final outcome of disease phenotypes cause by GSK3 activation is further modulated by other mechanisms, which are currently incompletely understood. Further studies are needed to examine how ALS-causing genes such as *SOD*, *TDP-43*, and *FUS* increase GSK3 activity in neurons and how GSK3 activation is involved in the cytoplasmic aggregate formation of toxic proteins.

PI3-K and its main downstream effector, Akt/protein kinase B, have been shown to play a central role in neuronal survival against apoptosis [64,65,66,67]. Interestingly, in the spinal cord of a SOD1^G93A^ mouse model of ALS, PI3-K and Akt expression decreased in a time-dependent manner [68]. Akt can inhibit GSK3β through the phosphorylation of GSK3β, indicating that altered GSK3β upstream signaling in SOD1^G93A^ mice may affect GSK3β activity (Figure 2). Semaphorin-3A (Sema3A), a member of the class 3 semaphorins, regulates the guidance of axonal and dendritic growth in the nervous system [69]. GSK3β regulates anterograde and retrograde dynein-dependent axonal transport in *Drosophila* and rats [70,71] and mediates Sema3A-induced axonal transport through the phosphorylation of Axin-1 [72]. It is also involved in Sema3A signaling through the motor neuron neuropilin-1 (NRP1) receptor to trigger distal axonopathy and muscle denervation in SOD1^G93A^ mice. Inhibition of Sema3A signaling via anti-NRP1 antibody restored the life span and rotarod motor function, reduced neuromuscular junction (NMJ) denervation, and attenuated ventral root pathology in the SOD1^G93A^ mouse model of ALS [73]. Insulin-like growth factor 2 (IGF-2), an activator of the PI3-K/Akt pathway, was maintained in oculomotor neurons in ALS and thus could play a role in oculomotor resistance in this disease. IGF-2 prolonged the survival of SOD1^G93A^ mice by preserving motor neurons and inducing nerve regeneration [74]. 

Recent studies have shown that chronic traumatic encephalopathy (CTE)-ALS is characterized by the presence of all six tau isoforms in both soluble and insoluble tau isolates. Activated GSK3β, pThr^175^ tau, pThr^231^ tau, and oligomerized tau protein expression were observed in hippocampal neurons and spinal motor neurons [75]. Other groups identified that genetic mutations in TDP-43 are associated with sALS and fALS [76,77]. A TDP-43-associated *Drosophila* ALS model, created by overexpression of wild-type human TDP-43 in *Drosophila* motor neurons, exhibited motor dysfunction and a dramatic reduction in life span [78,79]. The leg of an adult *Drosophila* is a valuable new tool for studying adult motor neuron phenotypes in vivo. Overexpression of TDP-43^Q331K^ in *Drosophila* motor neurons caused the progressive degeneration of adult motor axons and NMJs. Forward genetic studies have identified three genes that suppress TDP-43 toxicity, including *GSK3β*/*Shaggy*. Loss of *GSK3β*/*Shaggy* suppresses TDP-43^Q331K^-mediated axons and NMJ degeneration [80]. These results suggest that GSK3β plays a pivotal role in axonal degeneration in ALS. They also indicate that GSK3β is both directly and indirectly involved in the pathogenesis of ALS and could be a pharmacologically beneficial target for developing novel ALS treatments. However, the therapeutic potential of GSK3β-targeted drugs in patients with ALS remains uncertain, and further research is needed. 

To identify the therapeutic potential of GSK3β-targeted drugs in ALS treatment, many studies using in vivo models have been conducted. These studies have shown that GSK3β inhibitors can attenuate ALS disease progression. Lithium was the first natural GSK3β inhibitor to be identified, and it significantly increases the level of phosphorylated GSK3β serine 9, an inhibitory phosphorylation site [81,82,83,84]. Furthermore, lithium has been shown to have a neuroprotective role in neurodegenerative diseases [85]. Lithium treatment inhibits the Fas-mediated apoptosis signaling pathway, restores motor function defects, and inhibits disease progression in SOD1^G93A^ mice [86]. Valproic acid (VPA) is a well-known mood stabilizer, and its therapeutic effects in bipolar and affective disorders have been well studied. It also indirectly inhibits GSK3β via the regulation of Akt [87]. VPA acts as a neuroprotective agent for motor neurons, delays disease progression, and extends life span in SOD1^G93A^ mice [88]. Treatment with a combination of lithium and VPA exhibited greater and more consistent rescue effects on motor dysfunction and disease progression in the SOD1^G93A^ mouse model through the upregulation of phosphorylated GSK3β serine 9 levels compared to treatment with lithium or VPA alone [89]. Some studies have also shown that lithium treatment triggers autophagy and exhibits beneficial effects in ALS. For example, lithium treatment effects were concomitant with the activation of autophagy, increased the number of mitochondria in motor neurons of SOD1^G93A^ mice, and suppressed reactive astrogliosis [90]. However, the exact effect on autophagy activation by lithium remains unclear and the underlying mechanism has yet to be elucidated. GSK3 inhibitor VIII treatment markedly delayed the onset of symptoms, extended life span, and inhibited GSK3β activity in SOD1^G93A^ mice [91]. Furthermore, the inhibition of GSK3β activates HSF-1, which is associated with cell survival, reduction of cytochrome c release, caspase-3 activation, poly (ADP-ribose) polymerase (PARP) cleavage, and reduction of inflammation-related signals including cyclooxygenase-2 (COX-2) and intercellular adhesion molecule-2 (ICAM-2). Taken together, these findings suggest that GSK3β could be a potential target for developing treatments against ALS (Figure 2).

## 5. Studies Exploring the Role of GSK3β in In Vitro Models of ALS

Even with the availability of several excellent in vivo models of motor neuron degeneration for human ALS, in vitro models for the disease are still used in many studies, as they facilitate rapid screening of candidates for treatment. Several studies have investigated the effect of ALS on endoplasmic reticulum (ER)-mitochondrial signaling. Recent studies have shown that overexpression of TDP-43 or FUS leads to the activation of GSK3β, which regulates the endoplasmic reticulum (ER)–mitochondria association (Figure 2). It also perturbs ER–mitochondria interactions and cellular Ca^2+^ homeostasis in TDP-43- or FUS-overexpressed ALS models [92,93]. Other studies have shown that ALS with cognitive impairment (ALSci) is associated with tau phosphorylation at Thr^175^, which leads to the activation of GSK3β. This induces phosphorylation at tau Thr^231^ in HEK293T and Neuro2A cells [94]. In addition, IGF-2 induces Akt phosphorylation, GSK3β phosphorylation, and β-catenin levels while protecting ALS patient motor neurons [74]. Numerous studies have been conducted using in vivo and in vitro models to establish the therapeutic effect of GSK3β in ALS. Yang et al. found that kenpaullone had the ability to prolong the survival of motor neurons via the inhibition of GSK3β through small-molecule survival screening, using both wild-type and SOD1^G93A^ mouse embryonic stem cells [95]. Furthermore, kenpaullone also greatly improved the survival of human motor neurons derived from ALS-patient-induced pluripotent stem cells [95]. 

Vascular endothelial growth factor (VEGF) directly acts at the neuronal level as a potent neuroprotective agent against hypoxia and excitotoxicity [96,97,98,99]. VEGF treatment activates the PI3-K/Akt pathway and restores motor neuron cell death in SOD1^G93A^-transfected NSC34 cells [100]. VPA treatment also shows a protective effect in spinal motor neurons and protects them against death from glutamate toxicity [88]. Epigallocatechin gallate (EGCG), a major constituent of green tea polyphenols, is known to have protective effects against neurodegenerative diseases [101]. Multiple studies have suggested that EGCG affects numerous cell signaling pathways, including PI3-K/Akt, GSK3β, and caspase-3 [102]. EGCG treatment restores viability in oxidative-stress-induced cell death SOD1^G93A^-mutant cells by activating PI3-K/Akt and inhibiting GSK3β [103]. Studies have also shown that treatment with the GSK3β inhibitor 2-thio(3-iodobenzyl)-5-(1-pyridyl)-[1,3,4]-oxadiazole treatment in SOD1^G93A^-transfected VSC4.1 motor neuron cells increased their viability by activating HSF-1 and reducing cytochrome c release, caspase-3 activation, and PARP cleavage [104]. Taken together, these findings highlight the importance of GSK3β activity and function in the prevention of ALS.

## 6. Inhibitors of GSK3β in ALS Clinical Trials

GSK3β inhibitors are divided into five types depending on the mechanism. First, there are magnesium-competitive inhibitors that compete with magnesium, a cofactor of GSK3β, including lithium and zinc. Lithium inhibits GSK3β directly by competing with magnesium ions, and indirectly inhibits GSK3β by activating Akt [82,105]. The second are ATP-competitive inhibitors, such as indirubin [106] and meridianins [107], which act competitively on adenosine triphosphate (ATP), to prevent GSK3β from obtaining the phosphate it requires to phosphorylate target substrates. The third type are substrate-competitive inhibitors such as thiazolidinone [108], which binds to GSK3β instead of a substrate to prevent the phosphorylation of GSK3β target substrates. The disadvantage for substrate-competitive inhibitors is that they cannot pass through the blood–brain barrier (BBB) due to their high molecular weight. Fourth are modulators of GSK3β Ser9 phosphorylation that act by inhibiting enzymes, such as Akt, PKC, and Rsk1, to inhibit GSK3β Ser9 phosphorylation. For example, tamoxifen, an inhibitor of PKC, induces phosphorylation of GSK3β Ser9 to inhibit enzyme activity [109]. The final type are inhibitors that regulate protein binding in GSK3β, such as tamoxifen [110], which combines with GSK3β to control enzyme activity.

Of the many developed GSK3 inhibitors, only a few have reached clinical trials targeting human subjects and have been attempted in several neurodegenerative studies. For example, several clinical trials have evaluated the therapeutic effect of lithium on AD [111,112,113,114]. However, conflicting results were reported. Some studies reported no effects, whereas others reported mild therapeutic effects. Tideglusib, also known as NP-12 and NP031112, is a potent, selective small-molecule non-ATP-competitive GSK3β inhibitor. Preclinical studies have shown that tideglusib treatment results in reduced tau phosphorylation, neuronal loss, and rescued spatial memory deficits in transgenic mice [115]. Furthermore, in a phase IIa clinical trial in 30 patients with mild-moderate AD (NCT00948259), improvement in mini-mental status examinations and cognitive function was observed with tideglusib treatment [116]. In a subsequent phase IIb clinical trial of 308 patients with mild to moderate AD (NCT01350362), tideglusib proved safe in the trial, but development was discontinued to the lack of clinical benefits [117]. In the case of ALS, the first clinical study using lithium in ALS was reported in 2008 [90]. Lithium treatment delayed disease progression in ALS. This study included 44 patients. Remarkably, all patients treated with lithium were alive at the end of the study, whereas in the group receiving riluzole only, 29% of the subjects did not survive. These data indicate that patients with ALS, receiving lithium, progressed very slowly in the disease [90]. To date, many preclinical studies are actively underway, and these findings suggest that GSK3β can be a pharmacologically significant therapeutic target for the treatment of ALS. However, there are limitations on the use of GSK3β inhibitors. Further studies are needed to determine the precise role and molecular function of GSK3β in ALS pathogenesis.

## 7. Discussion

In the nervous system, GSK3 signaling plays critical roles in neurodevelopment and energy metabolism (Figure 1). Dysregulation of the GSK3 signaling pathway has been reported to be involved in most of the pathogenic mechanisms described for various neurodegenerative diseases, including AD, PD, and ALS. GSK3 has been demonstrated to promote several pathological phenotypes, such as Ab production and tau phosphorylation in AD. Growing evidence has been reported suggesting that GSK3 inhibition is effective in PD, AD, and ALS models. However, despite several experimental results, the contribution and physiological role of GSK3 in relation to disease pathology are still unclear. The GSK3 signaling pathway is a complex process influenced not only by cellular type but also by cellular conditions. In addition, GSK3 has been shown to be a kinase that is recruited to phosphorylate numerous substrates, which participate in various cellular processes. Therefore, a critical challenge in the field of neurodegenerative disease research is to understand how GSK3 participates in various signaling pathways and cellular processes. Therefore, it will be necessary to identify its genetic/physical partners or substrates and to understand their relative contribution to neuronal function, and as a consequence, their contribution to the pathogenesis of neurodegenerative diseases.

Abnormal activation of the GSK3 signaling pathway may promote pathological processes in various types of ALS models (Figure 2). In other words, suppressing high GSK3 activation may be sufficient to be beneficial for neuronal function. This raises the possibility that GSK3 inhibition may have an important effect on ALS pathogenesis. However, our knowledge of the exact biological and molecular functions of GSK3 in the pathogenesis of ALS caused by several RNA/DNA-binding proteins is still unclear, and further experiments are still necessary.

## 8. Conclusions

Data from several studies indicate that protein aggregation, proteasomal dysfunction, mitochondrial defects, neuroinflammation, and oxidative stress are involved in ALS pathogenesis. Therefore, understanding cellular signaling pathways, such as GSK3, which regulate neuronal dysfunctions linked to ALS pathogenesis, is important for the development of new strategies for ALS treatment.

GSK3β activity is increased in multiple ALS models and patients and has been associated with neuronal cell death in ALS. Furthermore, several studies have shown that GSK3β inhibition can rescue defective phenotypes of ALS in various models (Table 1). Recent studies have also revealed that RNA/DNA-binding proteins, such as TDP-43 and FUS, activate GSK3β, and that GSK3β inactivation suppresses TDP-43-induced neuronal toxicity. However, the following questions still need to be addressed in order to understand the exact neuroprotective mechanisms of GSK3β activity in the pathogenesis of ALS:

(1) How do ALS-causing genes, such as *SOD*, *TDP-43*, and *FUS*, increase the GSK3β activity in neurons?

(2) Which factors are critical for GSK3β activation in ALS?

(3) What is the effect of GSK3β inactivation on proteasomal dysfunction, neuronal toxicity, and mitochondrial dysfunction in ALS?

(4) Is GSK3β activation involved in the cytoplasmic aggregate formation of toxic proteins?

Addressing these questions will improve our understanding of ALS pathology and may provide valuable information about potential targets that can be used in the development of novel therapeutic drugs for treating ALS.

## Figures and Tables

**Table 1:** In vitro and in vivo studies exploring the role of GSK3β in ALS.

Type of Study	Model System	Key Findings	Reference
Ex Vivo	ALS patient sample (spinal cord, frontal and temporal cortices)	Increased GSK3β expression and cytosolic phospho-Y216 GSK3β in ALS patient sample	[61]
Ex Vivo	ALS patient sample (frontal and temporal cortices)	Increased GSK3β expression and cytosolic phospho-Y216 GSK3β in ALS patient sample	[62]
Ex Vivo	CTE-ALS patient sample (hippocampus and spinal cord)	Activated GSK3β, pThr175 tau, pThr231 tau, and oligomerized tau protein expression	[75]
In Vivo	SOD1^G93A^ transgenic mouse	Increased GSK3β activity via decreasing the PI3-K/Akt expression in an age-dependent manner	[68]
In Vivo	SOD1^G93A^ transgenic mouse	Inhibition of Sema3A/NRP1 signaling restored life span, motor function, and NMJ denervation	[73]
In Vivo	SOD1^G93A^ transgenic mouse	IGF-2 prolonged survival by preserving motor neurons and inducing nerve regeneration	[74]
In Vivo	TDP-43^Q331K^ transgenic fly	Loss of GSK3β/Shaggy suppressed TDP-43^Q331K^-mediated axon and NMJ degeneration	[80]
In Vivo	SOD1^G93A^ transgenic mouse	Treatment with lithium (a GSK3β inhibitor) improved neuron survival, motor function, and mortality	[86]
In Vivo	SOD1^G93A^ transgenic mouse	Treatment with VPA (a GSK3β inhibitor) rescued motor neuronal defects and delayed the disease progression	[88]
In Vivo	SOD1^G93A^ transgenic mouse	Treatment with a combination of lithium and VPA strongly rescued the motor dysfunction and disease progression via upregulation of phospho-S9 GSK3β	[89]
In Vivo	SOD1^G93A^ transgenic mouse	GSK3β inhibitor VIII treatment prolonged the life span via inhibition of GSK3β activity, preserved survival signals, and attenuated death and inflammatory signals	[91]
In Vitro	FUS/TDP43-transfected HEK293, NSC34 cells	ALS associated FUS, TDP-43 activated GSK3β to disrupt the VAPB–PTPIP51 interaction and ER–mitochondria associations	[92,93]
In Vitro	Tau-transfected HEK293T and Neuro2A cells	ALSci is associated with tau phosphorylation at Thr175 and leads to the activation of GSK3β, which induces phosphorylation at tau Thr231.	[94]
In Vitro	ALS-patients iPSC-derived motor neuron	IGF-2 induced Akt phosphorylation, GSK3β phosphorylation, and β-catenin levels while protecting ALS patient motor neurons	[74]
In Vitro	SOD1^G93A^ mESCs, ALS-patients iPSC-derived motor neuron	Kenpaullone treatment improved the survival of human motor neurons via inhibition of GSK3β	[95]
In Vitro	SOD1^G93A^-transfected NSC34 cells	GSK3β inhibitor: VEGF treatment activated PI3-K/Akt signaling and restored neuron cell death in motor neurons	[100]
In Vitro	PDC-induced mouse organotypic spinal cord	VPA (an inhibitor of GSK3β) treatment protected spinal motor neurons against glutamate toxicity	[88]
In Vitro	SOD1^G93A^-transfected VSC 4.1 cells	EGCG (an inhibitor of GSK3β) treatment restored viability in oxidative stress-induced cell death via activation of PI3-K/Akt signaling	[103]
In Vitro	SOD1^G93A^-transfected VSC 4.1 cells	2-thio(3-iodobenzyl)-5-(1-pyridyl)-[1,3,4]-oxadiazole (an inhibitor of GSK3β) treatment restored viability via activation of HSF-1 and reduction of cytochrome c release, caspase-3 activation, and PARP cleavage	[104]

CTE-ALS, chronic traumatic encephalopathy-amyotrophic lateral sclerosis; SOD1, superoxide dismutase 1; TDP-43, TAR DNA binding protein-43; FUS, fused in sarcoma; NMJ, neuromuscular junction; VPA, valproic acid; ER, endoplasmic reticulum; ALSci, amyotrophic lateral sclerosis cognitive inhibition; IGF-2, insulin-like growth factor-2; VEGF, vascular endothelial growth factor; EGCG, epigallocatechin gallate; HSF-1, heat shock transcription factor-1; PARP, poly (ADP-ribose) polymerase; VSC, ventral spinal cord.
